# Classification of Congeneric and QSAR of Homologous Antileukemic *S*–Alkylcysteine Ketones

**DOI:** 10.3390/molecules26010235

**Published:** 2021-01-05

**Authors:** Gloria Castellano, Adela León, Francisco Torrens

**Affiliations:** 1 Centro de Investigación Traslacional San Alberto Magno (CITSAM), Universidad Católica de Valencia San Vicente Mártir, Guillem de Castro-94, E-46001 València, Spain; 2 Escuela de Doctorado, Universidad Católica de Valencia San Vicente Mártir, E-46008 València, Spain; 3 Institut Universitari de Ciència Molecular, Universitat de València, Edifici d’Instituts de Paterna, P. O. Box 22085, E-46071 València, Spain

**Keywords:** partial correlation diagram, periodic classification, information entropy, principal component analysis

## Abstract

Based on a set of six vector properties, the partial correlation diagram is calculated for a set of 28 *S*-alkylcysteine diazomethyl- and chloromethyl-ketone derivatives. Those with the greatest antileukemic activity in the same class correspond to high partial correlations. A periodic classification is performed based on information entropy. The first four characteristics denote the group, and the last two indicate the period. Compounds in the same period and, especially, group present similar properties. The most active substances are situated at the bottom right. Nine classes are distinguished. The principal component analysis of the homologous compounds shows five subclasses included in the periodic classification. Linear fits of both antileukemic activities and stability are good. They are in agreement with the principal component analysis. The variables that appear in the models are those that show positive loading in the principal component analysis. The most important properties to explain the antileukemic activities (50% inhibitory concentration Molt-3 T-lineage acute lymphoblastic leukemia minus the logarithm of 50% inhibitory concentration Nalm-6 B-lineage acute lymphoblastic leukemia and stability *k*) are ACD log*D*, surface tension and number of violations of Lipinski’s rule of five. After leave-*m*-out cross-validation, the most predictive model for cysteine diazomethyl- and chloromethyl-ketone derivatives is provided.

## 1. Introduction

Nowadays, cancer is one of the most widespread diseases. It appears in different tissues and cells. Regarding its causes, there are a wide variety of carcinogens, both endogenous and exogenous. Breast, lung and colon are the most common cancers in developed countries. The global burden of cancer continues to increase, largely because of the aging and growth of the world population alongside the habits or behaviors that continuously expose us to carcinogens. Governments invest in preventive and informative public health campaigns. The most popular is against smoking, but there are many others such as preventives against breast and colon cancers, which are well known [1]. Owing to the rise in cancer, the search for anticancer drugs is still a target of study by many researchers. Most *S*-alkylcysteine diazomethyl- and chloromethyl-ketone derivatives have been shown to have anticancer action against acute lymphoblastic leukemia (ALL). They have been tested successfully [2,3,4,5,6,7]. The structures of these compounds are pretty close to amino acid cysteine (Cys).

The treatment of *N*-methoxycarbonyl *C*-carboxylate ester derivatives of *S*-methyl-l-cysteine by chloroperoxidase/hydrogen peroxide resulted in the oxidation of sulfur to produce (*R*_S_) sulfoxide in moderate to high diastereomeric excess [8]. The (*S*_S_) natural product sulfoxide chondrine was obtained via biotransformation of the *N*-*tert*-butyloxycarbonyl (Boc) derivative of l-4-*S*-morpholine-2-carboxylic acid using *Beauveria bassiana* or *B. caledonica*. The nucleophilic amino acids, largely employed for the peptide chemical modification, are the lysine and the cysteine residues. Cysteine modification is performed via its thiol side chain, which is characterized by a strong nucleophilicity, higher than that of a primary amine as amino acid lysine, which is protonated at pH values below 9.0. Therefore, a cysteine can react faster than lysine, resulting in the selective modification of a key amino acid over other residues. A possible synthetic route is the *S*-alkylation reaction; in this regard, post-translational modifications occurring on this amino acid are essential for the biological function of many proteins. In particular, numerous signaling proteins are post-translationally lipidated on a cysteine residue. Since this lipidation is essential for the correct localization and function of these proteins, the enzymes responsible for the covalent introduction/removal of lipid moieties have been considered interesting targets for blocking aberrant signaling processes [9].

In earlier publications, our research group showed a quantitative structure–activity relationship (QSAR) of sesquiterpene lactones (STLs) with potential antileukemic activity, with the aim of predicting inhibitors of Myb-induced gene expression and their mechanisms of action [10,11]. Moreover, molecular classifications of some series of phenolic compounds [12,13,14,15], triterpenoids and steroids [16] by information entropy were reported and related to their antioxidant activity. In the present report, 28 *S*-alkylcysteine diazomethyl- and chloromethyl-ketone derivatives were classified using this information entropy-based algorithm. The scientific rationale behind the classification is because the dodecyl derivative (**12**) is an exceptionally active compound against leukemia cells, the length of the alkyl chain has a profound effect on the antileukemic potency of the homologous series and the congeneric series may be useful for treating patients with therapy-refractory or relapsed leukemia. Thus, we want to validate if different moieties in the congeneric series correspond to the same potency. The objective of this study was to predict the antileukemic activity of these compounds based on their molecular structures; moreover, a study of QSAR and a principal component analysis (PCA) related the antileukemic activity of a homologous series of *S*-alkylcysteine chloromethyl-ketone derivatives to the physical and chemical properties of these compounds.

## 2. Results and Discussion

**Figure 1:**
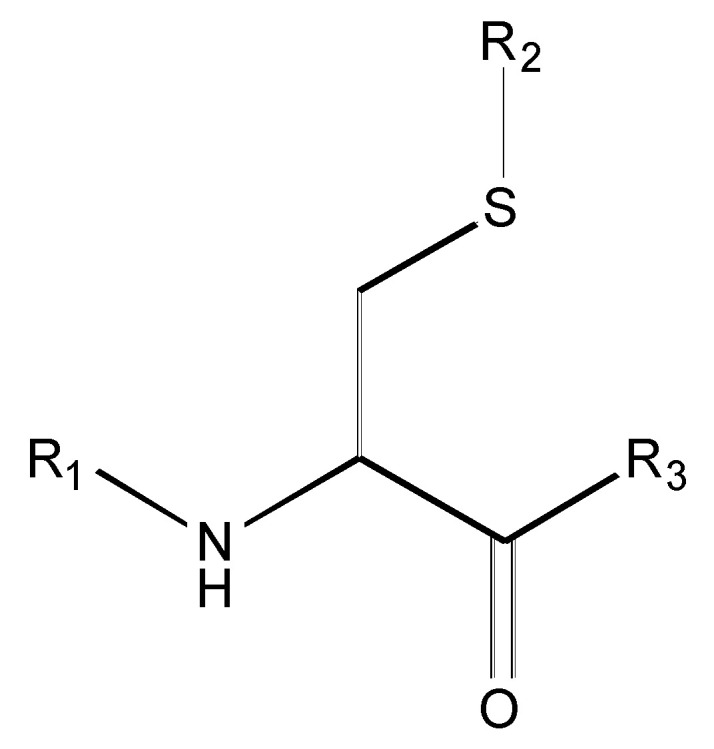
Basic structure of cysteine diazomethyl- and chloromethyl-ketone derivatives.

Figure 1 shows the basic structure of cysteine diazomethyl- and chloromethyl-ketone derivatives.

Table 1 lists the vector of properties of cysteine diazomethyl- and chloromethyl-ketone derivatives and experimental data of antileukemic activity (IC_50_) and stability *k*.

### 2.1. GraphCor Partial Correlation Diagram

**Figure 2:**
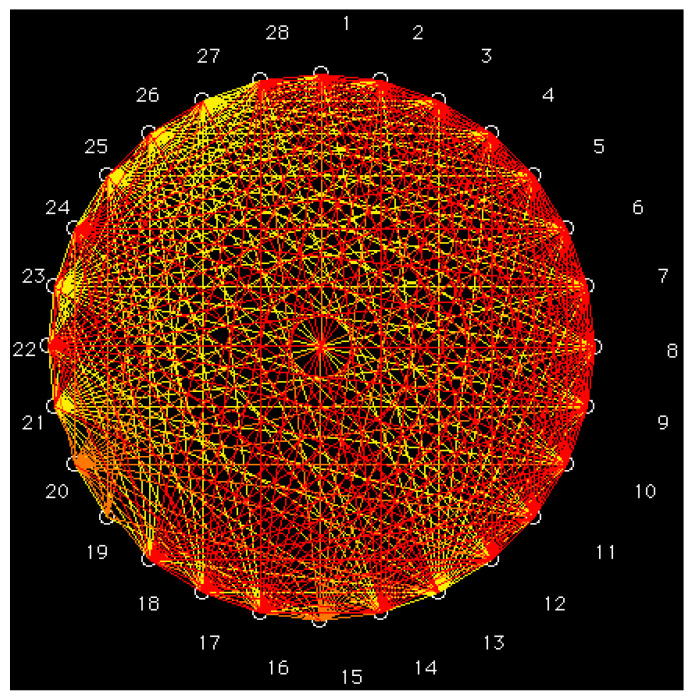
Partial correlation diagram: high (red), medium (orange) and low (yellow) correlations.

The matrix of Pearson correlation coefficients has been calculated between each pair of vector properties <*i*_1_,*i*_2_,*i*_3_,*i*_4_,*i*_5_,*i*_6_> for the 28 cysteine diazomethyl- and chloromethyl-ketone derivatives. The Pearson intercorrelations are computed for the partial correlation diagram, which contains high partial correlations (*r* ≥ 0.75), medium partial correlations (0.50 ≤ *r* < 0.75), low partial correlations (0.25 ≤ *r* < 0.50) and *zero* partial correlations (*r* < 0.25). Pairs of compounds with high partial correlation show a similar vector property. With the Equipartition Conjecture of Entropy Production, the partial correlations matrix (cf. Figure 2) contains 187 high, 44 medium, 116 low and 31 *zero* partial correlations. Many partial correlations are high. Red lines, representing high partial correlations, link cysteine derivatives with the greatest antileukemic activity because the most active compounds (**11** and **12**) are taken as reference molecules with vector properties <111111>. The antileukemic activities are expressed as IC_50_.

### 2.2. MolClas Molecular Classification Based on the Equipartition Conjecture of Entropy Production

The grouping rule is the following: two molecules are assigned to the same class if *r* ≥ *b*, where **b** is the classification level. A comparative analysis of the molecular dataset, from 28 classes (each compound in its own class) to one class (containing all compounds), by the method of information entropy theory, matching <*i*_1_,*i*_2_,*i*_3_,*i*_4_,*i*_5_,*i*_6_> and classification at level *b* (C_b_), is calculated for antileukemic activity [17] and summarized in Table 2.

The grouping rule in the case with equal weights *a*_k_ = 0.5, for the classification level 0.94 ≤ *b* ≤ 0.96, allows nine classes (grouped from Class 1 to Class 9, cf. Table 3).

**Figure 3:**
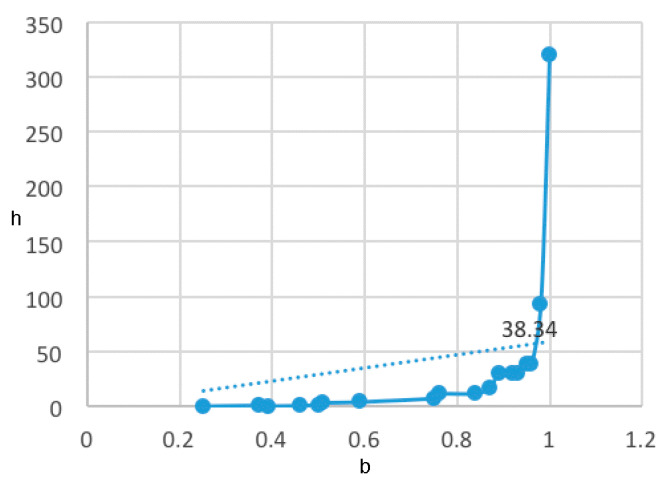
Entropy *h* vs. classification level *b* for different numbers of classes.

The classes above are obtained with the associated entropy *h*(**R**_b_) = 38.32, which is the classification closest to the cut-off point of the entropy vs. classification level with its trend line (cf. Figure 3).

Table 2 shows a classification of periodic properties by using a procedure based on the information entropy theory (artificial intelligence). The first four features were taken to denote the group or column, and the last two features were used to indicate the period or row in the table of periodic classification. Cysteine derivatives in the same group present similar properties. Furthermore, compounds also in the same period show maximum resemblance. In this report, the cysteine diazomethyl- and chloromethyl-ketone derivatives, in the table, are related to the experimental data of antileukemic bioactivity properties, taken from the technical literature [2,3,4,5,6,7]. The antileukemic activity increases on going right through a period and augments when descending in a group. The chloromethyl-ketone derivatives with the greatest activity (Class 1, compounds **11**, **12** and **24**) are grouped into the same class, corresponding to acetyl amides with a linear chain containing either 11 or 12 carbons in R_2_. Moreover, chloromethyl-ketone derivatives with great activity (Classes 2–5) are clustered into other groupings. Finally, the groups with the least antileukemic activity are cysteine diazomethyl derivatives and are located at the left side of the table (Classes 6–9). The results are in agreement with Figure 2 because pairs of compounds in the same class with similar vector properties <*i*_1_,*i*_2_,*i*_3_,*i*_4_,*i*_5_,*i*_6_> show red lines, representing high partial correlations, e.g., the pair (**11**, **12**) and both compounds with vector properties <111111> in Class 1.

### 2.3. Principal Component Analysis for Classification of the Most Antileukemic Bioactive Compounds

**Figure 4:**
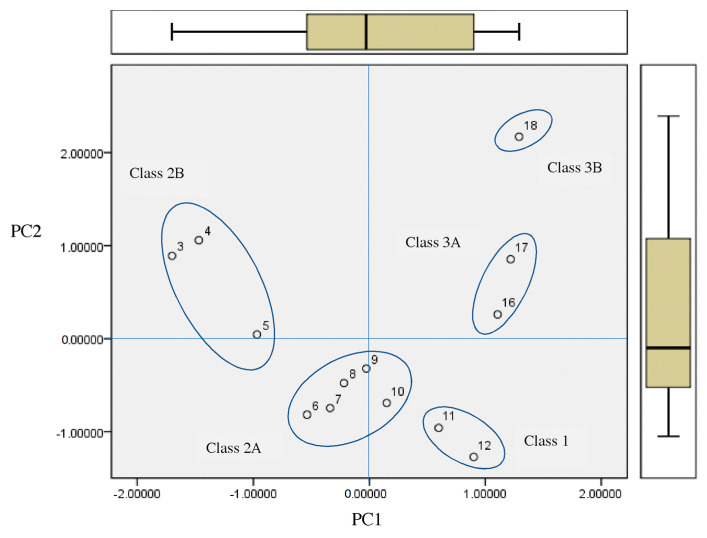
Scores plot for the homologous series of chloromethyl ketones with an acetyl group at R_1_.

After obtaining the classification of the cysteine chloromethyl- and diazomethyl-ketone derivatives, a PCA PC_1_–PC_2_ scores plot was made (cf. Figure 4) with the properties for the highly active compounds, forming a homologous series of chloromethyl-ketone derivatives with an acetyl group at R_1_ (compounds **3**–**12** and **16**–**18**). Compounds **1** and **2** are inactive, and neither are included because the value of stability *k* is not published for them. The following 18 properties were taken from the ChEMBL database and were used for statistical assessment: full molecular weight (Full_mw, *V*_1_), ACD log*P* (*V*_2_), number of rotatable bonds (rtb, *V*_3_), heavy atoms (*V*_4_), number of carbons in R_2_ (*V*_5_), a_logP (*V*_6_), boiling point (*V*_7_), enthalpy of vaporization (*V*_8_), a different estimation of ACD/logP (*V*_9_), molar volume (*V*_10_), polarizability (*V*_11_), ACD logD (pH 7.4, *V*_12_), ACD/KOC (pH 7.4, *V*_13_) ACD/BCF (pH 7.4, *V*_14_) Qed_weighted (*V*_15_), number of violations of Lipinski’s rule of five (Ro5, *V*_16_), surface tension (*V*_17_) and density (*V*_18_). In addition, the variables of both IC_50_ (µM) Nalm-6 B-lineage ALL (*V*_19_) and IC_50_ (µM) Molt-3 T-lineage ALL (*V*_20_), and stability *k* [hr^-1^] in 0.01M phosphate buffer, pH 7.5 (*V*_21_), were taken from the bibliographic experimental data of Uckun and coworkers [2,3,4,5,6,7]. Notice that there is only one entry for the logD value, compound **15** (chlorhydrate), different from logP; for the rest of them, there is no ionizable form, hence logP ~ logD for most of the compounds.

PCA was applied to reduce the initial variables to a small number of principal components (PCs) in order to obtain an overview variation of the compounds and identify behavioral patterns. Figure 4 shows the two-dimensional representation of the homologous series for all the variables taken into account for the first two PCs. The variance explained by PC_1_ and PC_2_ is 95.9%.

The homologous series of cysteine chloromethyl-ketone derivatives, with an acetyl group in R_1_, is distributed to five subclasses (Figure 4), in agreement with the clustering by entropy information and experimental data: Class 1 (compounds **11** and **12**, PC_1_ > 0, PC_2_ < 0, *bottom*), which includes the compounds with the greatest antileukemic activity, characterized by the presence of 11 or 12 carbons in R_2_; Class 2A (compounds **6**–**10**, PC_1_ < 0 in general, PC_2_ < 0, *middle*), characterized by the presence of 6–10 carbons in R_2_; Class 2B (compounds **3**–**5**, PC_1_ < 0, PC_2_ > 0, *left*), characterized by the presence of 3–5 carbons in R_2_; Class 3A (compounds **16** and **17**, PC_1_ > 0, PC_2_ > 0, *right*), characterized by the presence of 14 and 15 carbons in R_2;_ and Class 3B (compound **18**, PC_1_ > 0, PC_2_ >> 0, *top*), characterized by the presence of 16 carbons in R_2_. This scheme can be generalized to adopt a larger Class 3 merging Classes 3A and 3B.

**Figure 5:**
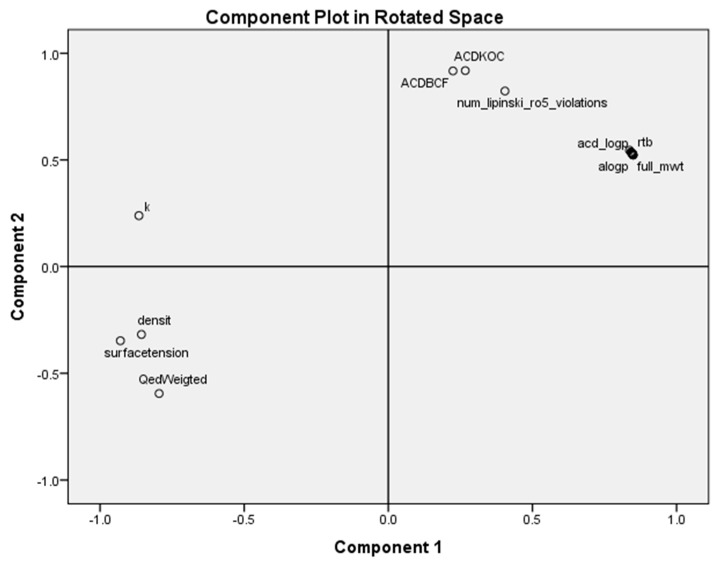
Loading plot of variables for homologous series of chloromethyl ketones with acetyl at R_1_.

Figure 5 describes the behavior of the variables. The properties most remote from the origin (0.0, 0.0) are the most important for describing PCs, and those closest to the origin are the least important.

On the one hand, PC_1_ (87.6% of the total variance) shows positive loading mainly with acd_logp, rtb, full_mwt, alogp, num_lipinski_ro5_violations, ACD/KOC and ACD/BDF, as well as negative loading with surface_tension, QedWeigted, density and stability *k*. On the other hand, principal PC_2_ (8.3% of the total variance) shows positive loading, mainly with ACD/BDF, ACD/KOC, num_lipinski_ro5_violations and *k*. The rest of the variables are near the origin and are less important for PC_2_.

Both compounds with important antileukemic activity and stability (**11** and **12**) are characterized by positive loading with the number of violations of Lipinski’s Ro5, ACD/KOC and ACD/BCF, as well as negative loading with surface tension, density and stability *k*. The rest of the variables are near the origin and are less important for antileukemic activity.

A multiple linear regression model approach was adopted to determine the quantitative importance of the combined presence of some of the 18 properties, taken from the ChEMBL database (*cf*. Supplementary Material Table S1) to explain such antileukemic activities: IC_50_ Molt-3 T-lineage ALL (higher value means lower antileukemic activity), pIC_50_ Nalm-6 B-lineage ALL (higher value means higher antileukemic activity) and stability *k* (higher value means higher antileukemic activity). The fits were checked with the correlation coefficient *r*, the standard deviation *s* and Fisher’s ratio *F*. The equations of the models between the homologous series of compounds and the properties follow:

(1) $$\begin{array}{l} IC_{50}\_Molt-3\_T-lineage\_ALL=-\left(653\pm 126\right)+\left(7.55\pm 1.29\right)ACD\log D\\ \;\;\;+\left(16.61\pm 3.19\right)surface\_tension \end{array}\;N=13\;r=0.895\;s=2.096\;F=20.1\;q=0.764$$

In the case of Nalm-6 B-lineage ALL, we have calculated pIC_50_ values because the *p* = −log function smoothens the data and provides a better correlation:

(2) $$\begin{array}{l} pIC_{50}\_Nalm-6\_B-lineage\_ALL=\left(187.3\pm 100.5\right)-\left(0.934\pm 0.427\right)Ro5\\ \;\;\;-\left(3.51\pm 1.79\right)surface\_tension \end{array}\;N=13\;r=-0.821\;s=0.270\;F=2.9\;q=0.424$$

(3) k=(0.0532±0.0063)−(0.00279±0.00120)ACDlogDN=13 r=−0.573 s=0.009 F=5.4 q=0.286

In Equation (1), the substitution of the dependent variable IC50_Molt-3_T-lineage_ALL by the pIC50 does not improve the correlation. The same occurs in Equation (3) for the substitution of *k* by log*k*. All the results are in agreement with the PCA (Figure 5) because both IC_50_ variables show positive loading, among others, with ACD log*D*, surface tension and number of violations of Lipinski’s Ro5.

Quantitative structure–activity/property relationship (QSAR/QSPR) researchers are trying to establish equations that correlate the physicochemical parameters of the molecules with their activities/properties; e.g., molar refractivity, refractive index and electronic parameters, which have been used extensively. The first study that correlated the surface tension with dissociation constants was Thakur’s work [18]. He showed that the surface tension could be successfully used to model the dissociation constant of sulfonamide drugs. The dissociation constant p*K*_a_ depends on the polarity and the intermolecular forces. For maximum activity, the sulfonamides should present a proper p*K*_a_ for penetrating in vivo membranes and best binding abilities to their target enzyme. The abilities depend on their protonated/unprotonated form dissociation constants, expressed as p*K*_a_. Thakur’s results could explain the interest of surface tension appearing in Equations (1) and (2) because it reduces the bioactivity of our molecules.

**Figure 6:**
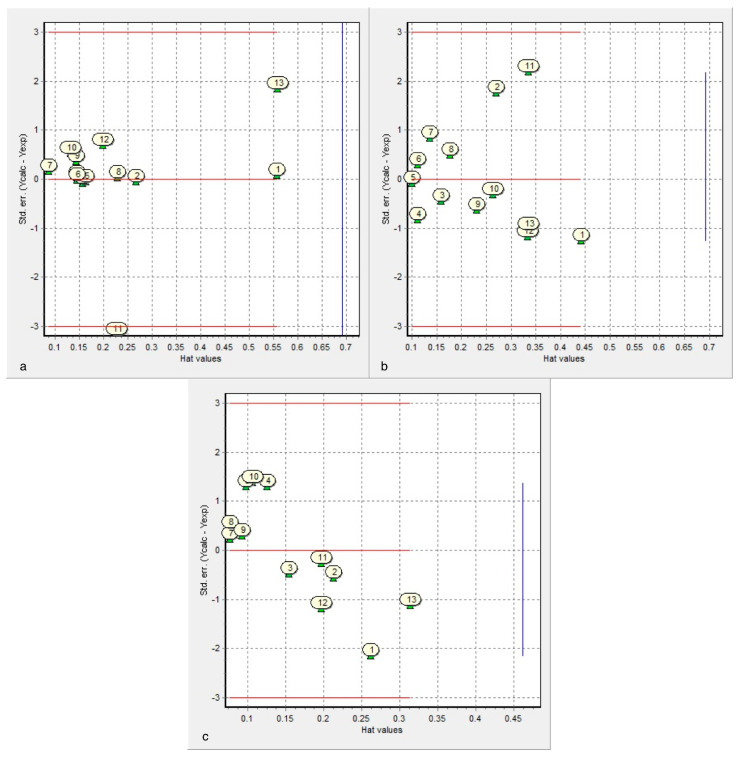
The Williams plot for the graphical visualization of outliers for the response (on the *Y*-axis: standardized residuals >3σ) or for the structure (on the *X*-axis: highest Hat value >h* cut-off line) in the regression models: (**a**) Equation (1); (**b**) Equation (2); (**c**) Equation (3). Numbers 1–13 correspond to compounds **3**–**12**, **16**–**18**.

The applicability domain of the proposed models (1)–(3) is analyzed by Williams plot (*cf*. Figure 6), which is the chart of cross-validated standardized residuals vs. leverage (Hat diagonal) values (*k*). In Equation (1), the response outlier (cross-validated standardized residual >3σ) is compound **16** and the structurally influential chemical (*h* > h*) is compound **18**. In Equation (2), there is neither response outlier nor structurally influential chemical. In Equation (3), there is no response outlier and the structurally influential chemical is compound **18**.

Leave-*m*-out (1 ≤ *m* ≤ 10) cross-validated correlation coefficients *r*_cv_ calculated for Cys diazomethyl- and chloromethyl-ketone derivatives (*q* = *r*_cv_ (*m* = 1), cf. Table 4) show that *r*_cv_ decays with *m* except for IC_50_ Molt-3 T-lineage and pIC_50_ Nalm-6 B-lineage (Equations (1) and (2)), which indicates possible outliers. In Equation (2), cross-validation can be calculated for only *m* ≤ 2 because Ro5 values are not very discriminating (cf. Table S1). In particular, the Molt-3 T-lineage activity inhibition model IC_50_ vs. {ACDlogD, surface_tension (Equation (1)) gives the greatest *r*_cv_. Therefore, Equation (1) results more predictive than Equations (2) and (3).

**Figure 7:**
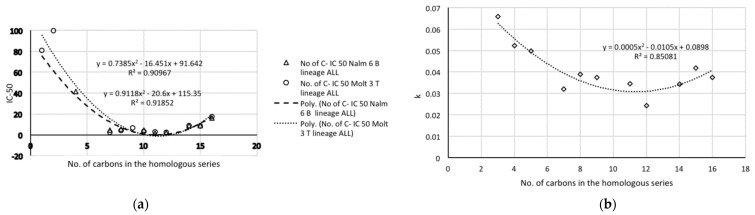
Experimental data: (**a**) IC_50_ antileukemic activity and (**b**) stability *k* vs. the number of carbons.

The linear regressions suggest that the number of carbons is an important individual factor. Figure 7a,b shows the representations of both IC_50_ Nalm-6 B-lineage ALL and IC_50_ Molt-3 T-lineage ALL, as well as stability *k* vs. the number of carbons. Both IC_50_ Nalm-6 and IC_50_ Molt-3 are similar, with the minimum in 11-12 carbon atoms (Figure 7a). All properties are fitted to second-degree polynomial curves. The most active compounds (**11** and **12**), which present minimum values in the fitted models in the graphics, match Class 1 in Table 2 of periodic properties, obtained by the procedure based on information entropy theory (artificial intelligence). These compounds are in the last (right side) group and last (bottom) period.

**Figure 8:**
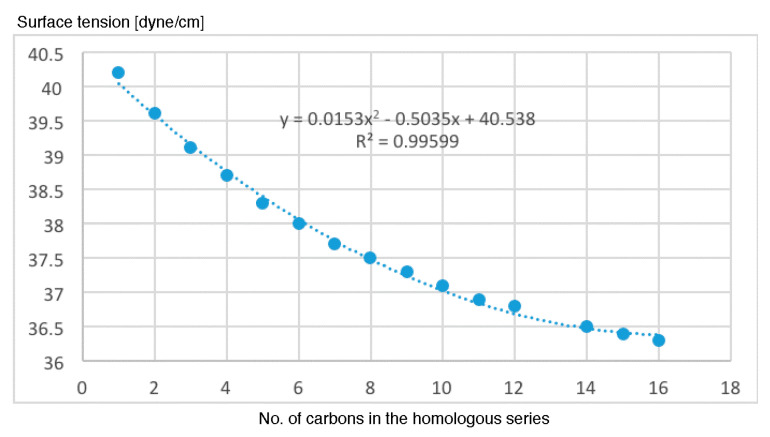
Surface tension data vs. the number of carbons.

Figure 8 displays surface tension data vs. the number of carbons for the homologous series. The surface tension decays monotonically with the number of carbons.

## 3. Materials and Methods

### 3.1. MolClas Program for Molecular Classification Based on the Equipartition Conjecture of Entropy Production

The computational method is the same as the one that we successfully applied to the classification of polyphenolic compounds. The first step in quantifying the concept of similarity, for molecules of cysteine diazomethyl- and chloromethyl-ketone derivatives, is to list the most important moieties with respect to the antileukemic activity of such compounds. Furthermore, the vector of properties $\bar{i}$ = <*i*_1_,*i*_2_,…*i*_k_,…> should be associated with each feature *i*_k_, whose components correspond to a number of characteristic functional groups in the molecule, in a hierarchical order, according to the expected importance of their antileukemic activity. The components *i*_k_ are either “1” or “0,” according to the experimental conclusions of antileukemic power for structural variations in the cysteine derivative compounds.

In this way, index *i*_1_ = 1 denotes a chloromethyl group at R_3_; *i*_2_ = 1 signifies either an acetyl or *tert*-butyloxycarbonyl (Boc)-substituent at R_1_; *i*_3_ = 1 indicates the only presence of an acetyl group at R_1_; *i*_4_ = 1 means a chain that has between 3 and 12 carbons in line either with or without ramifications, either with or without double bonds at R_2_; *i*_5_ = 1 represents that at R_2_; the structure presents a chain with either 11 or 12 carbons in line, either with or without ramifications and either with or without double bonds; and *i*_6_ = 1 shows the absence of ramifications and double bonds in the R_2_ chain (Table 1).

Let us denote by *r_ij_* (0 ≤ *r_ij_* ≤ 1) the similarity index of two cysteine derivatives, associated with the $\bar{i}$ and $\bar{j}$ vectors, respectively. A similarity matrix R = [*r_ij_*] characterizes the relation of similitude. The similarity index between two cysteine derivatives $\bar{i}$ = <*i*_1_,*i*_2_,…*i_k_*,…> and $\bar{j}$ = <*j*_1_,*j*_2_,…*j_k_*,…> is defined as:

(4) $$r_{ij}=\sum_{k}t_{k}{\left(a_{k}\right)}^{k}\;(k=1,2,\ldots )$$

where 0 ≤ *a_k_* ≤ 1 and *t_k_* = 1 if *i_k_* = *j_k_*, but *t_k_* = 0 if *i_k_* ≠ *j_k_*. This definition assigns a weight (*a_k_*)*^k^* to each property involved in the description of molecule *i* or *j*. The hierarchical order of the six structural features is expressed by their corresponding weights. For instance, for all *a_k_* = 0.5, these weights are 0.5, 0.25, 0.125, 0.0625, 0.03125 and 0.015625, which have been used in this work.

Learning procedures similar to those encountered in stochastic methods are implemented as follows [19]. Consider a given partition into classes as good or ideal from practical or empirical observations. This corresponds to a reference similarity matrix **S** = [*s_ij_*] obtained for an arbitrary number of fictitious properties. Next, consider the same set of species as in the good classification and the actual properties.

The similarity degree *r_ij_* is then computed from the **R** correlation matrix. The number of properties for **R** and **S** may differ. The learning procedure consists of trying to find classification results for **R** as close as possible to the good classification. The distance between the partitions in classes characterized by **R** and **S** is given by:

(5) $$D=-\sum_{ij}\left(1-r_{ij}\right)\ln\frac{1-r_{ij}}{1-s_{ij}}-\sum_{ij}r_{ij}\ln\frac{r_{ij}}{s_{ij}}\;\forall 0\leq r_{ij},s_{ij}\leq 1$$

This definition was suggested by that introduced in information theory by Kullback to measure the distance between two probability distributions [20]. Such a procedure has been applied to the synthesis of complex dendrograms using information entropy [21,22].

We have written a MolClas program for molecular classification based on the Equipartition Conjecture of Entropy Production. It punches the similarity and difference matrices, as well as the latter in format NEXUS (.NEX) for programs PAUP, MacClade and SplitsTree. Code MolClas performs single- and complete-linkage hierarchical cluster analyses (CAs) of the compounds by using the IMSL subroutine CLINK [23].

### 3.2. GraphCor Program for Partial Correlation Diagram

The partial correlation diagram presents high partial correlations (|r| ≥ 0.75) in red, medium partial correlations (0.50 ≤ |r| < 0.75) in orange, low partial correlations (0.25 ≤ |r| < 0.50) in yellow and *zero* partial correlations (|r| < 0.25) in black. Codes MolClas and GraphCor are available from the authors at Internet (torrens@uv.es) and are free for academics.

### 3.3. Statistical Analysis

Principal component analysis (PCA), linear and multiple linear regression models were performed using SPSS (vs. 21.0, IBM Corp., USA), Minitab (vs. 17.1.0), Knowledge Miner and Microsoft Excel for Office (2020) for Windows 10 OS. The calculated statistics are the number of data points *N*, the correlation coefficient *r*, the standard deviation *s* and the Fisher’s ratio *F*. The correlation coefficients between cross-validation *r*_cv_ (*q* = *r*_cv_ (*m* = 1), etc.) were calculated with the leave-*m*-out (LMO) procedure [24]. The process furnishes a new method for selecting the best set of descriptors: LMO selects the best set of descriptors according to the criterion of maximization of the value of *r*_cv_. The cross-validation was used to determine the predictability of the models, which were compared and validated taking into account *r*_cv_ (*q*).

## 4. Conclusions

From the discussion of the present results, the following conclusions can be drawn.

1. Based on a set of six vector properties, the partial correlation diagram was calculated for a set of 28 *S*-alkylcysteine diazomethyl- and chloromethyl-ketone derivatives. Derivatives with the greatest antileukemic activity in the same class correspond to high partial correlations.
2. A table of periodic classification is made based on information entropy. The first four characteristics denote the group, and the last two indicate the period. Nine classes are clearly distinguished. The most active compounds (**11**, **12** and **24**), all with 11 or 12 carbons in line in R_2_, are situated at the right side, bottom and, especially, bottom right of this periodic table.
3. The principal component analysis scores plot of the homologous series of *S*-alkyl chloromethyl ketones, for 18 properties, shows five subclasses corresponding to the periodic classification of the congeneric series into nine classes.
4. Linear fits of both antileukemic activities and stability are good (correlation coefficients of 0.57 or greater). They are in agreement with the principal component analysis. The variables that appear in the models are those that show positive loading in the principal component analysis.
5. The most important properties to explain the antileukemic activities (50% inhibitory concentration Molt-3 T-lineage acute lymphoblastic leukemia minus the logarithm of 50% inhibitory concentration Nalm-6 B-lineage acute lymphoblastic leukemia and stability *k*) are ACD log*D*, surface tension and number of violations of Lipinski’s rule of five.
6. After leave-*m*-out cross-validation, Equation (1) is the most predictive for cysteine diazomethyl- and chloromethyl-ketone derivatives (cross-validated correlation coefficient of 0.764).
7. The results of the antileukemic activities for the cysteine diazomethyl- and chloromethyl-ketone derivatives show that the surface tension has an unfavorable influence and this could be related to the results obtained by Thakur.
8. The representations of 50% inhibitory concentration Nalm-6 B-lineage and 50% inhibitory concentration Molt-3 T-lineage acute lymphoblastic leukemias, as well as stability *k* vs. the number of carbons, are fitted to second-degree polynomial curves. The most active compounds (**11** and **12**) present minimum values and coincide with Class 1 obtained by information entropy theory.

## Figures and Tables

**Table 1:** Vector of properties of Cys diazo- and chloromethyl-ketone derivatives and experimental data of antileukemic activity (IC_50_) and stability *k*.

Compound	R_1_	R_2_	R_3_	<*i*_1_,*i*_2_,*i*_3_,*i*_4_,*i*_5_,*i*_6_> ^a^	IC_50_ (µM) Nalm-6 B-lineage ALL	IC_50_ (µM) Molt-3 T-lineage ALL	*k* [hr^−1^] 0.01M Phosphate Buffer, pH = 8.0, Ionic Strength = 0.3 M
**1**	CH_3_CO	CH_3_	CH_2_Cl	111001	30.3	80.8	–
**2**	CH_3_CO	CH_2_CH_3_	CH_2_Cl	111001	52.8	99.9	–
**3**	CH_3_CO	(CH_2_)_2_CH_3_	CH_2_Cl	111101	6.9	8.0	0.0658
**4**	CH_3_CO	(CH_2_)_3_CH_3_	CH_2_Cl	111101	41.4	5.6	0.0523
**5**	CH_3_CO	(CH_2_)_4_CH_3_	CH_2_Cl	111101	5.8	5.4	0.0498
**6**	CH_3_CO	(CH_2_)_5_CH_3_	CH_2_Cl	111101	3.3	0.7	0.0336
**7**	CH_3_CO	(CH_2_)_6_CH_3_	CH_2_Cl	111101	4.8	2.5	0.0319
**8**	CH_3_CO	(CH_2_)_7_CH_3_	CH_2_Cl	111101	5.6	4.1	0.0388
**9**	CH_3_CO	(CH_2_)_8_CH_3_	CH_2_Cl	111101	7.3	6.7	0.0373
**10**	CH_3_CO	(CH_2_)_9_CH_3_	CH_2_Cl	111101	4.7	3.4	0.0352
**11**	CH_3_CO	(CH_2_)_10_CH_3_	CH_2_Cl	111111	1.7	3.0	0.0345
**12**	CH_3_CO	(CH_2_)_11_CH_3_	CH_2_Cl	111111	2.0	2.3	0.0242
**13**	CH_3_CO	(CH_2_)_11_CH_3_	CH=N_2_	011111	15.4	22.9	–
**14**	Boc ^b^	(CH_2_)_11_CH_3_	CH_2_Cl	110111	15.1	15.5	–
**15**	H ^c^	(CH_2_)_11_CH_3_	CH_2_Cl	100111	17.7	12.5	–
**16**	CH_3_CO	(CH_2_)_13_CH_3_	CH_2_Cl	111001	8.7	8.8	0.0417
**17**	CH_3_CO	(CH_2_)_14_CH_3_	CH_2_Cl	111001	8.9	8.6	0.0374
**18**	CH_3_CO	(CH_2_)_15_CH_3_	CH_2_Cl	111001	16.0	17.3	0.0363
**19**	Boc-Gly	*trans,trans*-Farnesyl	CH=N_2_	000110	51.3	84.5	–
**20**	Boc-Gly	*trans,trans*-Farnesyl	CH_2_Cl	100110	12.9	17.5	–
**21**	Boc	*trans,trans*-Farnesyl	CH=N_2_	010110	49.8	50.1	–
**22**	Boc	*trans,trans*-Farnesyl	CH_2_Cl	110110	10.7	7.7	–
**23**	CH_3_CO	*trans,trans*-Farnesyl	CH=N_2_	011110	30.3	32.2	–
**24**	CH_3_CO	*trans,trans*-Farnesyl	CH_2_Cl	111110	3.0	1.4	–
**25**	CH_3_CO	*trans*-Geranyl	CH=N_2_	011000	>100	>100	–
**26**	Boc	*trans*-Geranyl	CH=N_2_	010000	>100	>100	–
**27**	CH_3_CO	3-Methyl-2-butenyl	CH=N_2_	011000	>100	>100	–
**28**	CH_3_CO	3-Methyl-2-butenyl	CH_2_Cl	111000	12.6	7.9	–

^a^ i_1_ = 1, a chloromethyl group at R_3_; i_2_ = 1, either an acetyl or Boc-substituent at R_1_; i_3_ = 1, the only presence of an acetyl group at R_1_; i_4_ = 1, a chain with between 3 and 12 carbons in line either with or without ramifications, either with or without double bonds at R_2_; i_5_ = 1, at R_2_, a chain with either 11 or 12 carbons in line, either with or without ramifications, either with or without double bonds; i_6_ = 1, absence of ramifications and double bonds in the R_2_ chain. ^b^ Boc: tert-butyloxycarbonyl. ^c^ The molecule is a hydrochloride (acid salt resulting from its reaction with hydrochloric acid).

**Table 2:** Classification of cysteine diazomethyl- and chloromethyl-ketone derivatives by information entropy method.

P ^a^	0001 ^b^	0100/0101/0110	0111	1001	1101	1110	1111
0X ^c^		**Class 9**				**Class 3**	**Class 2**
		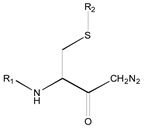				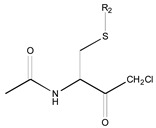	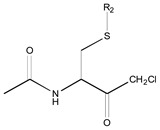
		**25** R_1_: CH_3_CO; R_2_: *trans*-Geranyl **26** R_1_: Boc; R_2_: *trans*-Geranyl **27** R_1_: CH_3_CO; R_2_: 3-Methyl-2-butenyl				**1** R_2_: -CH_3_ **2** R_2_: -CH_2_CH_3_ **16** R_2_: -(CH_2_)_13_CH_3_ **17** R_2_: -(CH_2_)_14_CH_3_ **18** R_2_: -(CH_2_)_15_CH_3_ **28** R_2_: 3-Methyl-2-butenyl	**3** R_2_: -(CH_2_)_2_CH_3_ **4** R_2_: -(CH_2_)_3_CH_3_ **5** R_2_: -(CH_2_)_4_CH_3_ **6** R_2_: -(CH_2_)_5_CH_3_ **7** R_2_: -(CH_2_)_6_CH_3_ **8** R_2_: -(CH_2_)_7_CH_3_ **9** R_2_: -(CH_2_)_8_CH_3_ **10** R_2_: -(CH_2_)_9_CH_3_
1X	**Class 8**	**Class 7**	**Class 6**	**Class 5**	**Class 4**		**Class 1**
	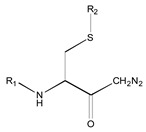	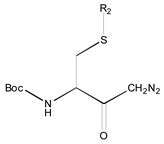	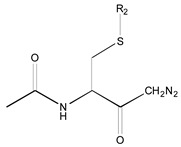	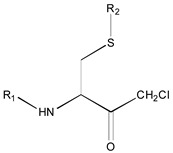	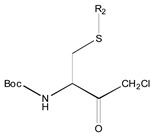		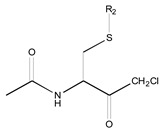
	**19** R_1_: Boc-Gly; R_2_: *trans,trans*-Farnesyl	**21** R_2_: *trans,trans*-Farnesyl	**13** R_2_: -(CH_2_)_11_CH_3_ **23** R_2_: *trans,trans*-Farnesyl	**15** R_1_: H.HCl; R_2_: -(CH_2_)_11_CH_3_ **20** R_1_: Boc-Gly; R_2_: *trans,trans*-Farnesyl	**14** R_2_: -(CH_2_)_11_CH_3_ **22** R_2_: *trans,trans*-Farnesyl		**11** R_2_: -(CH_2_)_10_CH_3_ **12** R_2_: -(CH_2_)_11_CH_3_ **24** R_2_: *trans,trans*-Farnesyl

^a^ P: period <*i*_5_,*i*_6_>. ^b^ 0001: group <*i*_1_,*i*_2_,*i*_3_,*i*_4_>. ^c^ X = either 0 or 1.

**Table 3:** Entropy and classification level *b* for different numbers of classes.

*b*	*h*	No. of Classes
1.0000	320.8858	28
0.9799	93.3938	14
0.9599	38.3400	9
0.9499	38.3178	9
0.9299	30.4859	8
0.9199	30.5388	8
0.8899	30.5166	8
0.8699	17.4259	6
0.8399	11.8925	5
0.7599	11.5383	5
0.7499	7.5860	4
0.5899	4.1698	3

**Table 4:** Cross-validated correlation coefficient in leave-*m*-out for Cys diazomethyl- and chloromethylketones.

*m*	IC_50_ Molt-3 T-Lineage ALL Equation (1)	pIC_50_ Nalm-6 B-Lineage ALL Equation (2)	*k* Equation (3)
1	0.764	0.424	0.286
2	0.767	0.428	0.285
3	0.770	–	0.283
4	0.772	–	0.281
5	0.775	–	0.280
6	0.776	–	0.280
7	0.775	–	0.283
8	0.769	–	0.290
9	0.738	–	0.306
10	–	–	0.340

## Data Availability

Data is contained within the article or supplementary material.

## Supplementary Materials

Supplementary materials can be found online.

## Sample Availability

The samples of compounds are not available from authors.
