# Supplementary material for: Classification of Congeneric and QSAR of Homologous Antileukemic S–Alkylcysteine Ketones
Source: Molecules. 2021 Jan 5;26(1):235. doi: 10.3390/molecules26010235 (PMC7795853; doi:10.3390/molecules26010235)
Supplement: Supplementary file 1 [file molecules-26-00235-s001.pdf]

Table TS1. Properties or variables of the homologous series of chloromethyl-ketone derivatives with an acetyl group at R_1 and a chloromethyl group at R_3 taken from ChEMBL database.

Cmpn.	full mwt	Qed weighted	acd logp	num Lipinski Ro5 vltns	alogp	rtb	Heavy atoms	Density [g/cm^3]	Boiling point [°C] at 760mmHg	Enthalpy of vaporizatior [kJ/mol]	ACD Log*P*	ACD/ Log*D* (p*H* 7.4)	ACD/ BCF (p*H* 7.4)	ACD/ KOC (p*H* 7.4)	Polarizabilit × 10^{24} [cm^3]	Surface tension [dyne/cm	Molar volume [cm^3]	No. of C in R
**1** 357349	209.70	0.68	-0.15	0	0.66	5	12	1.2	405.2	65.7	1.17	0.79	2.36	64.26	20.2	40.2	172.9	1
**2** 357820	223.72	0.69	0.36	0	1.05	6	13	1.2	412.7	66.5	1.70	1.13	4.28	98.56	22.0	39.6	189.4	2
**3** 150541	237.75	0.54	0.87	0	1.44	7	14	1.2	421.4	67.5	2.23	1.58	9.27	171.37	23.9	39.1	205.9	3
**4** 150509	251.78	0.53	1.38	0	1.83	8	15	1.1	430.9	68.6	2.76	2.04	20.76	305.12	25.7	38.7	222.4	4
**5** 149917	265.81	0.51	1.89	0	2.22	9	16	1.1	441.1	69.8	3.29	2.71	67.12	706.75	27.5	38.3	238.9	5
**6** 152904	279.83	0.49	2.40	0	2.61	10	17	1.1	451.6	71.1	3.83	3.14	144.31	1222.44	29.4	38.0	255.4	6
**7** 149915	293.86	0.47	2.91	0	3.00	11	18	1.1	462.4	72.3	4.36	3.69	376.21	2427.15	31.2	37.7	271.9	7
**8** 155452	307.89	0.44	3.42	0	3.39	12	19	1.1	473.3	73.6	4.89	4.19	901.08	4535.43	33.0	37.5	288.4	8
**9** 346198	321.91	0.41	3.93	0	3.78	13	20	1.1	484.4	75.0	5.42	4.94	3321.09	11537.90	34.9	37.3	304.9	9
**10** 153126	335.94	0.38	4.44	0	4.17	14	21	1.1	484.4	75.0	5.42	4.94	3321.09	11537.90	34.9	37.1	304.9	10
**11** 153179	349.97	0.35	4.95	0	4.56	15	22	1.0	506.5	77.6	6.48	5.71	12944.47	30550.50	38.6	36.9	337.9	11
**12** 540205	364.00	0.31	5.46	0	4.95	16	23	1.0	517.6	79.0	7.01	6.07	24159.48	47752.92	40.4	36.8	354.4	12
**16** 153041	392.05	0.24	6.48	1	5.73	18	25	1.0	539.5	81.7	8.07	7.35	225458.66	236211.23	44.1	36.5	387.5	14
**17** 153047	406.08	0.21	6.99	1	6.12	19	26	1.0	550.4	83.0	8.61	7.88	577181.63	462933.16	45.9	36.4	404.0	15
**18** 347764	420.10	0.18	7.50	1	6.51	20	27	1.0	561.2	84.4	9.14	8.39	1000000.00	875320.88	47.7	36.3	420.5	16

Cmpn..: compound (boldface) and Chemical Abstract Service (CAS) number.
hba Lipinski = 3 in all cases.
hbd Lipinski = 1 in all cases.
Polar surface area = 71.00Å^2 in all cases.
psa = 46.17Å^2 in all cases.
